# Bumping Into Doors: A Case Report of Brain Abscess as a Complication of Pulmonary Arteriovenous Malformation

**DOI:** 10.7759/cureus.88315

**Published:** 2025-07-19

**Authors:** Sylvain Pilate, Daan Van Olmen, Ilse Sergant, Frederik Vandereyken

**Affiliations:** 1 Department of Emergency, AZ Klina, Brasschaat, BEL; 2 Department of Emergency, Ziekenhuis Geel, Geel, BEL

**Keywords:** brain abscess, case report, embolization, hereditary hemorrhagic telangiectasia, neurosurgery, pulmonary arteriovenous malformation

## Abstract

Cerebral abscess is an infection of the central nervous system that can cause a variety of neurologic signs. The majority of infections are caused by distant haematogenous spread, neurotrauma, surgery, or contiguous infections. Another rare source of a brain abscess is a pulmonary arteriovenous malformation (PAVM). Late diagnosis and neurological complications associated with brain abscesses can cause significant morbidity or even mortality.

We present the case of a 75-year-old Caucasian male with complaints of subfebrillitas, headache, and difficulty in estimating distances. A diagnosis of brain abscess was made. The abscess was drained, and ceftriaxone plus metronidazole was initiated. After the cultures were positive for *Streptococcus anginosus* and *Fusobacterium*, the therapy was de-escalated to penicillin G and metronidazole. In search of a focus, a PAVM was identified and subsequently embolised.

Brain abscess is a relatively rare condition. The prevalence of PAVMs is also considered uncommon. The majority of cases is linked to hereditary haemorrhagic telangiectasia (HHT), an autosomal dominant hereditary disease, but PAVMs can also occur as a single-standing problem. Neurological events such as cerebral abscess, stroke, or migraine may occur in some cases of PAVMs. To prevent complications, embolisation of every radiographically visible PAVM needs to be considered. Moreover, PAVMs should be considered in the search for the aetiology of a brain abscess when routine investigations fail to detect other causes. The coexistence of HHT in every patient with PAVMs also needs to be considered.

## Introduction

Cerebral abscess is an infection of the central nervous system that can cause a variety of neurologic signs. Most infections are caused by distant haematogenous spread, neurotrauma, surgery, or contiguous infections such as otitis media and sinusitis. In 15%-30% of patients, no source is found. Less common causes of brain abscesses include right-to-left shunts, such as a patent foramen ovale or, as in our case, a pulmonary arteriovenous malformation (PAVM). PAVMs are rare congenital vascular malformations that form a direct connection between the pulmonary artery and vein, causing an anatomic right-to-left shunt. The majority of PAVMs are linked to hereditary haemorrhagic telangiectasia (HHT). Neurological complications associated with PAVMs include brain abscesses or recurrent strokes, which can cause significant morbidity or even mortality. In our case report, we describe a symptomatic brain abscess as a rare complication of a PAVM. We will discuss the aetiology of PAVMs, neurological complications, and treatment options. 

This article was previously presented by the authors as a meeting abstract at the 2021 BESEDIM Congress on January 30, 2021 [1].

## Case presentation

A 75-year-old Caucasian man was referred to our Emergency Department (ED) by his general practitioner. The patient had a 10-day history of headache and low-grade fever. The complaints gradually worsened over four days but then stabilised. At presentation, the headache was negligible, whereas fatigue and persistent low-grade fever were his main concerns. Further anamnesis revealed that the patient had difficulty estimating distances, which led to him bumping into doors multiple times at home. No other neurological symptoms were present. There were no signs of coughing, dyspnoea, or anosmia. 

His medical history revealed basal cell carcinoma of the nose. There was no use of chronic medication. Clinical examination revealed a blood pressure of 150/80 mmHg, a heart rate of 85 bpm, an oxygen saturation of 95% without supplemental oxygen, and a respiratory rate of 14 breaths per minute. At presentation, his body temperature was 38°C. Auscultation of the lungs and heart was normal, and there were no signs of meningism or lateralisation. Further examination showed a homonymous quadrant anopsia of the right inferior quadrant. Laboratory results showed an elevated C-reactive protein (CRP) level of 10.9 mg/dL, with a normal white blood cell count and neutrophil count. Afterwards, the result of the COVID-19 polymerase chain reaction (PCR) test turned out to be negative (Table 1).

**Table 1:** Lab results The bold number is indicated as an abnormal value. CRP, C-reactive protein; PCR, Polymerase chain reaction

	Lab results	Unit	Reference range
CRP	10.9	mg/dL	< 0.5 mg/dL
White blood cells	7.56	x10^3^/µL	3.45 - 9.76 x 10^3^/µL
Neutrophils	61.1	%	40.2 - 74.7%
COVID-19 PCR	Negative	-	-

**Figure 1:**
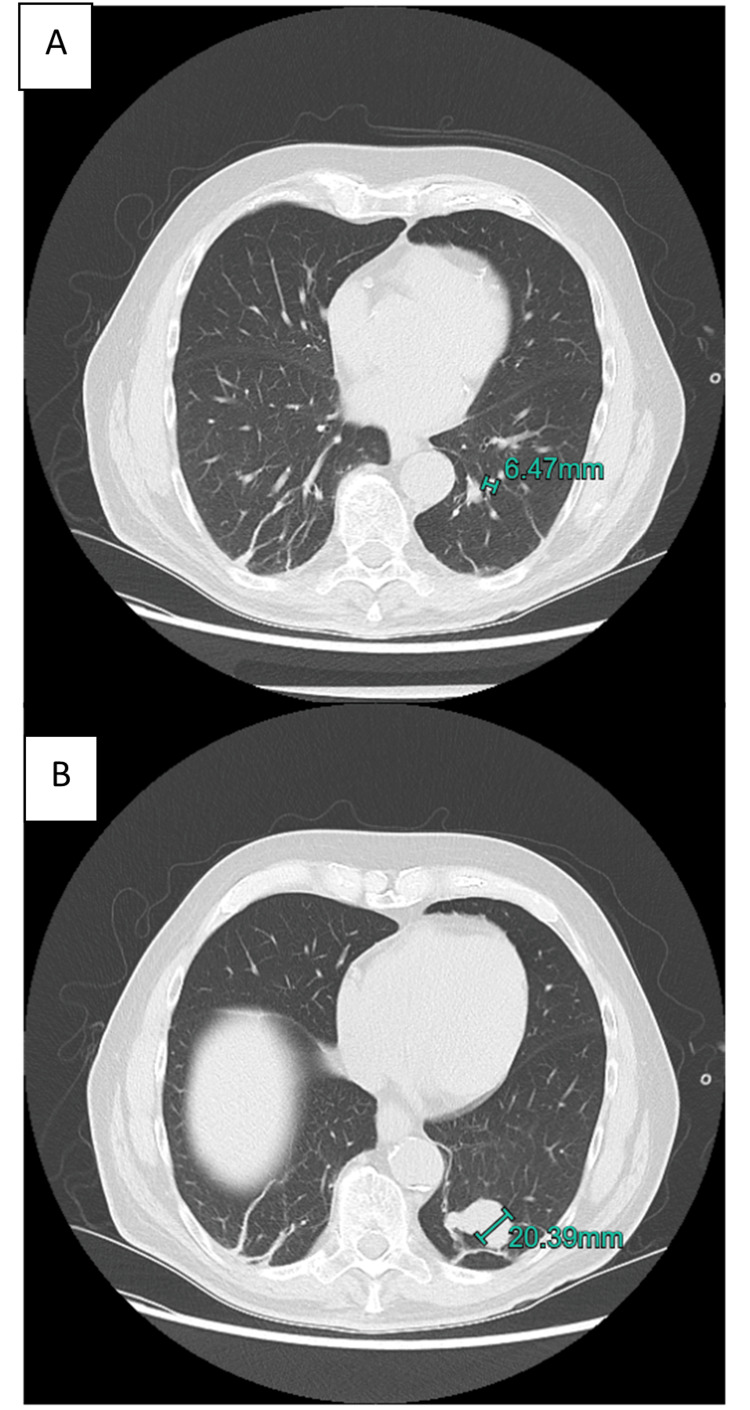
CT with intravenous contrast of the chest showing a pulmonary AVM A) Supplying artery measuring 6.47 mm; B) AVM with a maximum diameter of 20.39 mm. AVM, Arteriovenous malformation; CT, Computed tomography

Computed tomography (CT) of the brain was performed immediately and showed a cystic lesion of 38 mm in the left occipital lobe, with perilesional oedema, suspected to be a metastasis or glioblastoma. In search of a primary malignancy, CT with intravenous contrast of the chest and abdomen was carried out on the same day, which showed no suspicious malignant lesions. However, an incidental arteriovenous malformation (AVM) was found in the left inferior lobe of the lung, with an arterial diameter of 6.47 mm (Figure 1). 

**Figure 2:**
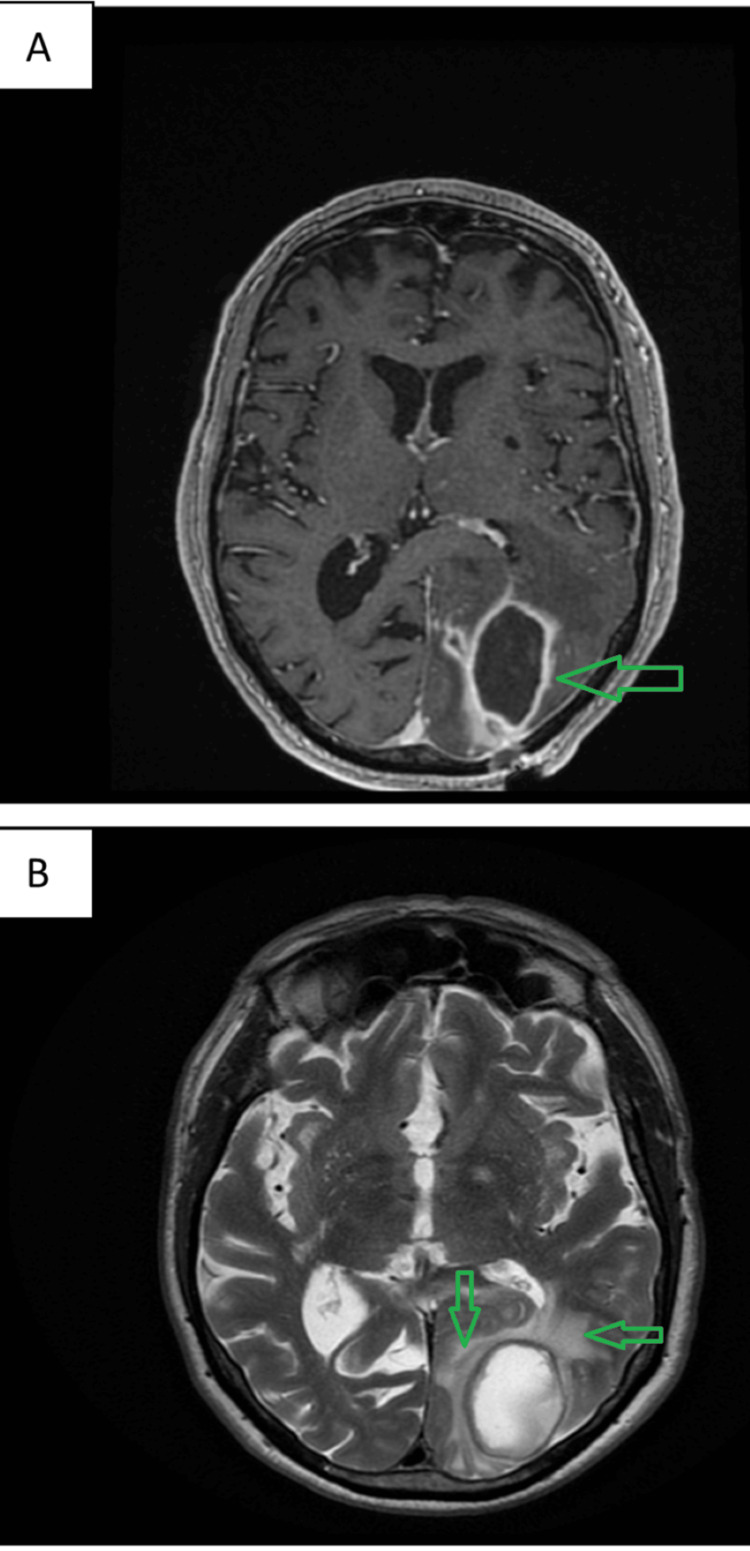
MRI of the brain A) T1 with gadolinium, showing a ring-shaped contrast enhancement suggestive of a brain abscess (arrow); B) T2 showing perilesional oedema (arrows). MRI, Magnetic resonance imaging

The patient was hospitalised at our Neurology Department. To differentiate the cystic lesion in the brain, magnetic resonance imaging (MRI) was carried out one day later, and it was suggestive of an underlying brain abscess (Figure 2).

The brain abscess was drained two days after presentation at our ED. A sample for culture and anatomopathological investigation was taken, and empiric intravenous antibiotic therapy with 2 g of ceftriaxone once a day and 1.5 g of metronidazole once a day was initiated. Anatomopathological examination of the sample showed no signs of malignancy, but a large amount of neutrophils compatible with an abscess. After the cultures tested positive for *Streptococcus anginosus* and *Fusobacterium*, antibiotic therapy was de-escalated to benzathine penicillin G - six times 4,000,000 units intravenously per day - and metronidazole intravenously, 1.5 g once a day, for four weeks based on the present antibiogram. Intravenous therapy was changed to peroral therapy after four weeks: amoxicillin (1 g) three times a day and metronidazole (500 mg) three times a day for another four weeks.

MRI of the abscess 10 days after drainage showed a slight decrease in the volume of the abscess and the formation of a daughter abscess; therefore, a total resection of the lesion was performed. Most likely, the initial drainage was not carried out completely successfully.

**Figure 3:**
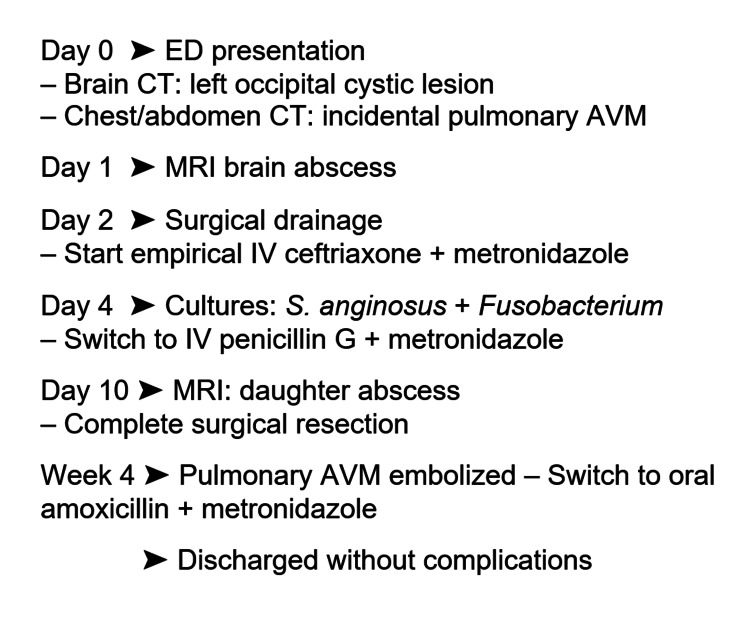
A flow diagram of the clinical course ED, Emergency department; CT, Computed tomography; MRI, Magnetic resonance imaging; AVM, Arteriovenous malformation

A work-up to identify the underlying focus of the brain abscess was performed. There was no dental focus, nor prostatitis, and transthoracic echocardiography (TTE) of the heart showed no patent foramen ovale or signs of endocarditis. TTE revealed a right-to-left shunt compatible with the diagnosed PAVM. The AVM was embolised three weeks after presentation, and the patient was discharged after one month without any complications. A flow diagram of the clinical course is presented in Figure 3.

## Discussion

Cerebral abscess is a relatively rare condition that affects approximately 0.3 to 0.9 per 100,000 people per year [2,3]. Moreover, rates are increased in immunocompromised patients [3,4]. Brain abscesses are commonly caused by distant haematogenous spread infections (endocarditis, bacteraemia, etc.), trauma, neurosurgery, or adjacent infections such as otitis media, dental infections, and sinusitis [2,3,5]. In 15%-30% of cases, no source of infection can be found, and these cases are classified as cryptogenic brain abscesses [5,6]. Another, more uncommon, distant focus for brain abscess is a cardiac right-to-left shunt, such as a patent foramen ovale, cyanotic cardiac disease, or, as in our case, a PAVM.

Aetiology of PAVMs

PAVMs are abnormal vascular connections between pulmonary arteries and veins that cause an anatomic right-to-left shunt. Due to the increasing use of CT in medical practice, the prevalence of PAVMs is approximately 1 in 2,600 people [7]. Most PAVMs are linked to HHT, an autosomal dominant hereditary disease affecting approximately 1-5 in 8,000 people. HHT is caused mainly by mutations in ENG (HHT1), ACVRL1/ALK1 (HHT2), or Smad4 (HTJP). The incidence of PAVMs in HHT patients ranges from 18% to 58%, depending on the causal genotype [7]. HHT-related PAVMs are mostly multiple in number, and fewer than 40% of cases involve a single AVM [5]. On the other hand, the aetiology of PAVMs can be caused by factors other than HHT. In contrast to HHT-related PAVMs, the fistulas are more commonly single AVMs (80%) and are mostly asymptomatic, detected by routine CT [5]. PAVMs in this category may arise from earlier operations for congenital heart disease, trauma, or underlying hepatopulmonary syndrome. In other cases, as in our case, isolated PAVMs can be found in the absence of congenital heart disease, trauma, or surgery. Some of them are present at birth and thus congenital, but other AVMs are detected later in life and might not have been present at the beginning of life [7,8]. The origin of PAVMs is the subject of further genetic investigation in HHT, but there also seems to be a role for hepatic factors in stimulating or inhibiting pulmonary angiogenesis [8].

Complications

PAVMs are characterised by an anatomic bypass of the arteriovenous capillary bed, causing defective filter function. Under normal conditions, the capillary bed acts as a filter to prevent bacteria or small thrombi from entering the systemic circulation. The most feared complication of PAVMs is neurological impairment, which is observed in approximately 35% (range 5%-59%) of patients [5,6]. Many neurologic events due to PAVMs are poorly recognised, and in recent series, a median delay from cerebral event to PAVM diagnosis was observed to be two years [7]. The majority of patients with secondary neurological diseases were not aware of their diagnosis of PAVMs.

The neurological complications described below can be explained by the mechanism of a paradoxical embolism passing across the PAVM. Symptoms are more likely to occur when the diameter of the feeding artery is greater, or when multiple PAVMs are present [5].

The incidence of brain abscess in patients with PAVMs/HHT is 390-fold greater than that in the healthy population: 155 per 100,000 per year compared with 0.4 per 100,000 per year [9]. Most of the organisms associated with PAVM-related brain abscesses are anaerobic. Male sex and recent dental interventions were identified as risk factors for PAVM-related brain abscess [7].

In addition to brain abscess, another important neurological complication is the presence of (recurrent) ischaemic stroke. The lifetime risk of stroke related to PAVM differs between 9% and 18% [7]. Cerebral ischaemia occurs in 14%-32% of patients with a single PAVM. However, this number increases by 60% in patients with multiple PAVMs [10]. Risk factors associated with ischaemic stroke include high fibrinogen levels and low serum iron levels. Iron deficiency, as a cause of blood loss in HHT, contributes to higher blood viscosity and impaired oxygen delivery. On the other hand, most PAVM-related strokes are not global ischaemic events but rather focal anterior or posterior syndromes. More recent studies have shown that platelets from iron-deficient patients are more likely to aggregate to serotonin, which could be an important starting point for new therapeutic agents [7,11]. In addition to embolisation, the use of antiplatelet aggregation is recommended for patients with stroke due to PAVMs, even if there is a greater risk of bleeding in patients with HHT [12].

Studies suggest that embolisation of PAVMs reduces migraine headaches in patients with HHT. The risk of migraine in HHT patients is doubled if these patients have PAVMs. A plausible explanation for the link with migraine is the production of vasoactive amines secondary to modified pulmonary metabolism [7]. Other, more frequent, non-neurologic complications of PAVMs include dyspnoea, haemorrhage, chest pain, and myocardial infarction.

Treatment

According to the latest guidelines of the British Thoracic Society and a recent Cochrane review publication, all patients with radiographically visible PAVMs must be referred to a centre where interventional embolisation can be performed [11,13]. In the past, only PAVMs with a diameter greater than 3 mm were embolised [5]. Nevertheless, new studies suggest that every PAVM detected by CT should be embolised, independent of the diameter of the feeding artery [11].

Embolisation of PAVMs reduces the risk of paradoxical embolisms and other complications. On the other hand, there was a resolution of the right-to-left shunt, causing an improvement in oxygenation in patients with multiple PAVMs [6,11]. Complications of embolisation are rare (1%) but should be considered [11]. The most common complication is transient pleuritis, but rare complications, such as massive haemoptysis or paradoxical embolism of devices, air bubbles, or thrombi, have also been described [7]. An individual patient-guided and multiprofessional approach is recommended. Post-embolisation follow-up with CT is advised because recanalisation or new PAVMs can develop. Embolisation has replaced the surgical approach, but surgery continues to play an important role in urgent haemorrhage control or in PAVMs that cannot be treated with embolisation.

## Conclusions

PAVMs should be considered when determining the aetiology of a brain abscess or ischaemic stroke, especially when routine investigations fail to detect other causes. Embolisation reduces the risk of paradoxical embolism and thus must be considered in every radiographically detected PAVM. In all patients with PAVMs, the coexistence of HHT must be considered. Given the relationship with HHT, our proposal is to screen for HHT, as serious complications - such as cerebral AVMs - in the patient and his relatives can be prevented with early detection of these AVMs.
